# Exploring Risk Factors Related to Low Calf Circumference in Older Adults With Multimorbidity: Cross-Sectional Latent Class Analysis

**DOI:** 10.2196/68760

**Published:** 2025-10-02

**Authors:** Xilin Peng, Xudong Chen, Ruihao Zhou, Fanfan Shi, Tao Zhu, Guo Chen

**Affiliations:** 1 Department of Anesthesiology, National Clinical Research Center for Geriatrics, West China Hospital, Sichuan University, No. 37 Guoxue Lane, Wuhou District, Chengdu, Sichuan, 610041, China, 86 18980601577; 2 The Research Units of West China (2018RU012)–Chinese Academy of Medical Sciences, West China Hospital, Sichuan University, Chengdu, Sichuan, China; 3 Department of Clinical Research and Management, Center of Biostatistics, Design, Measurement and Evaluation (CBDME), West China Hospital, Sichuan University, Chengdu, Sichuan, China

**Keywords:** low calf circumference, multimorbidity, sarcopenia, older adults, China

## Abstract

**Background:**

As the global population continues to age, the prevalence of sarcopenia is gradually increasing, and the loss of skeletal muscle mass is one of the manifestations of sarcopenia. Low calf circumference (CC) is often used as a predictor of poor skeletal muscle mass or sarcopenia. Older adults usually have a combination of multiple chronic diseases. There is a lack of evidence to explore the risk factors for low CC with multimorbidity in Chinese, community-dwelling, older adults.

**Objective:**

This study aimed to explore the risk factors and potential categories in older adult patients with low CC and multimorbidity from an individual-centered perspective.

**Methods:**

We selected 15,874 participants from the Chinese Longitudinal Healthy Longevity Survey in 2018 and screened for low CC in older adult patients. The individual-centered latent class analysis was used to classify potential multimorbidity groups. Multiple logistic regression was used to explore the risk factors associated with low CC and multimorbidity by applying the elastic net to screen for reliable risk variables.

**Results:**

A total of 7956 older individuals were eligible for the study, of whom 3960 (49.8%) were aged >90 years and 2166 (27.2%) had multimorbidity with low CC. The prevalence of multimorbidity increases between the ages of 65 and 89 years. However, the majority of older adults remain in reasonably good health beyond the age of 90 years. Five multimorbidity groups were identified by latent class analysis: multisystem morbidity diseases (78/2166, 3.6%), arthritis-rheumatism or rheumatoid diseases (400/2166, 18.47%), diabetes-hypertension diseases (330/2166, 15.23%), respiratory-heart diseases (347/2166, 16.02%), and cardiovascular diseases (1011/2166, 46.68%). Through 12 variables screened by the elastic net, multiple logistic regression showed different impacts on multimorbidity groups, including demographic background, behavioral characteristics, and physical and mental health factors. In particular, older patients who self-report poor health and live in urban areas need more attention.

**Conclusions:**

The results revealed that low CC is a common phenomenon among community-dwelling older adults, and a substantial proportion also present with multimorbidity. In the older adult population with low CC, the proportion of multimorbidity does not simply increase with age. Multimorbidity in low CC has been identified in 5 potential groups. Different groups have distinctive risk factors. Public health authorities should pay attention to low CC in older adult patients with multimorbidity and carry out targeted interventions, thereby enhancing health outcomes.

## Introduction

### Background

It is projected that approximately 200 million individuals worldwide will develop sarcopenia by 2050 [1]. Muscle fiber degeneration typically begins around the age of 50 years, with up to 50% of fibers diminished by the age of 80 years. Notably, this decline is also observed in physically active individuals, including athletes [2]. Calf circumference (CC) is a commonly used measurement to assess lower limb anthropometrics; it outperforms BMI or waist circumference and exhibits moderate to high sensitivity and specificity in predicting sarcopenia or poor skeletal muscle mass [3,4]. According to the Asian Working Group for Sarcopenia 2019 guidelines, low CC can be used to identify patients with sarcopenia in advance among older adult individuals in China. Increasing evidence suggests that low CC is capable of detecting patients with sarcopenia across various physical conditions [5,6]. In addition, the research found that low CC was associated with disability in people aged >60‐70 years [7] and could even predict the mortality risk among older adults in both the United States and Taiwan [8,9].

The prevalence of type 2 diabetes, cerebrovascular diseases, cancer, and neurodegenerative disorders significantly increases with age [10]. Older adults often have more than one chronic disease. Multisystem disorders can shorten life expectancy and exacerbate already existing imbalances in the distribution of medical resources [11]. Approximately 42.4% of people in China aged >50 years live with multiple diseases [12]. Nearly 70% of all fatalities in China are attributed to the nation’s rapidly aging population and the high prevalence of chronic illnesses [12]. Studies have confirmed that multimorbidity is not a simple random combination but has some underlying physiological and pathological connections [13]. Identifying the combination of different categories in multimorbidity will be beneficial in achieving an individual-centered medical care pattern and improving the clinical outcomes of patients with multimorbidity.

The evidence from UK Biobank participants suggests a link between probable sarcopenia and long-term conditions as well as multimorbidity [14]. A study identified 5 distinct multimorbidity patterns associated with lower limb muscle loss in China [15]. However, there is a lack of data to explore the relationship between older adults with low CC and multimorbidity, which is essential for the management of healthy aging.

### Objectives

Our study aimed to investigate the epidemiology of older adults with low CC and multimorbidity in the Chinese community, including potential multimorbidity categories, and to explore related risk factors, thereby establishing the theoretical foundation for implementing prevention and management measures to achieve healthy aging in the older adult population with low CC and multimorbidity.

## Methods

### Participants

The Chinese Longitudinal Healthy Longevity Survey (CLHLS) is a comprehensive and representative longitudinal study on the health and lifespan of older people in China. Following a baseline survey conducted in 1998, the CLHLS conducted 7 waves of surveys every 2 to 3 years from 2000 to 2018, covering 22 of China’s 31 provinces and approximately 85% of the population. The study used the CLHLS 2018 data, which included a total of 15,874 older adult participants. This is in accordance with the design of our study, includes an effective sample size, and reflects the most recent health behaviors of older adults in China.

### Ethical Considerations

The Peking University Research Ethics Committee authorized the CLHLS study (IRB00001052-13074), and all participants or proxy respondents gave written informed consent. Each respondent who agreed to participate in the survey signed a consent form confirming that they were aware of the objective of the research. All processes were executed following the relevant rules and standards. The CLHLS data used in our study were completely anonymized and deidentified. All personal identifiers were removed from the dataset and replaced with unique identification codes that cannot be traced back to individuals. The dataset contains no personally identifiable information, such as names, detailed addresses, or ID numbers. According to Article 32 of the Ethical Review Measures for Life Science and Medical Research Involving Human Beings issued by China in 2023, scientific research based on legally obtained anonymized public data, which does not involve biological samples and does not violate the commercial interests of others, can be exempted from ethical review. As the CLHLS followed strict protocols to protect participant confidentiality and privacy, no second ethical review was conducted for our study, and no additional informed consent was obtained due to the anonymity of the participants. Further, based on available public documentation about the CLHLS, there is no explicit information regarding specific compensation provided to participants. The survey primarily focused on collecting data about older adult health and living conditions to support academic research and policy development.

### Calf Circumference

CC (measured in centimeters) was rounded up to an integer in the CLHLS 2018 wave. Trained investigators measured CC with participants seated, with their hips and knees bent at 90° and their feet flat on the floor. A nonelastic tape was placed around the calf without compressing the tissue and moved along its length to identify the maximum circumference. Each leg was measured twice, and the average was recorded. The final value was the mean of both legs [16]. According to the content of the 2018 questionnaire, CC was rounded to the whole number and entered into the dataset. All investigators were strictly trained and followed standard operating procedures. Additional quality control details are available on the CLHLS website. The diagnosis of our study adopted the cutoff of low CC advised by Asian Working Group for Sarcopenia 2019: CC<34 cm for men and CC<33 cm for women [17]. These cutoffs were derived from multicenter cohorts of community-dwelling older adults in Asia to support standardized sarcopenia screening in primary care and public health.

### Multimorbidity

Every individual was asked if they had experienced any of the following 17 chronic diseases: hypertension, heart disease, bronchitis, emphysema, pneumonia or asthma, tuberculosis, gastric or duodenal ulcer, dyslipidemia, cholecystitis or cholelith disease, chronic nephritis, hepatitis, stroke or cardiovascular disease (CVD), dementia, epilepsy, Parkinson disease, arthritis, rheumatism or rheumatoid disease, cancer, and diabetes. The 17 chronic diseases were selected from the CLHLS questionnaire by excluding sex-specific conditions and retaining those most representative of chronic disease patterns among older Chinese adults, based on previous literature [18,19]. The co-occurrence of at least 2 chronic diseases in 1 person is referred to as multimorbidity [20].

### Multidimensional Risk Factors

On the basis of certifications from previous studies [21,22], we identified relevant risk variables that represent the characteristics of China’s older adult population. Demographic data included age, sex, place of residence, marital status, and educational level. Behavioral characteristics consisted of current smoking status, current alcohol consumption, physical activity, and sleep quality. No physical activity implies that the participants do not engage in hobbies such as reading, watching television, listening to the radio, or making friends, among other things. Physical and psychological health characteristics included scores from the Mini-Mental State Examination (0‐30 points) for the cognitive function test, Center for Epidemiological Studies-Depression Scale 10-item (CES-D-10; 0‐30 points), and Generalized Anxiety Disorder 7-item (GAD-7; 0‐21 points) in the past 2 weeks; additional measures included fall experience in the past year, BMI, self-reported health, and difficulty with activities of daily living (ADLs). ADLs included 6 items: bathing, dressing, simple indoor activities (getting in and out of bed or sitting up), eating, using the toilet, and controlling urination and defecation. Difficulty with at least 1 of the abovementioned items was classified as a *yes*. All variables were collected by trained interviewers through standardized questionnaires as part of the CLHLS survey, ensuring data quality and consistency.

### Statistical Analysis

#### Descriptive Statistics

The normality of continuous variables was checked using the Kolmogorov-Smirnov test. Continuous variables with normal distributions were represented by means and SDs, while those with abnormal distributions were represented by medians and IQRs. Categorical variables and frequencies (%) were used. Two-tailed independent sample *t* tests, Mann–Whitney U tests, and chi-square tests were used, as appropriate, to compare the baseline characteristics between participants with multimorbidity and those who were relatively healthy. Moreover, we used the MissForest algorithm for missing value imputation. MissForest effectively imputes missing values in multidimensional and mixed continuous and categorical data. This method has lower imputation errors and no parameter adjustment or data distribution assumptions [23].

#### Latent Class Analysis

Latent class analysis (LCA) has been widely used in health behavior research to categorize latent features based on apparent behaviors and identify the proportion of each class to execute intervention approaches for each subclass. The 17 types of multimorbidity were answered with a yes or no response (1 or 0). The Akaike information criterion (AIC), Bayesian information criterion (BIC), adjusted BIC (aBIC), Lo-Mendell-Rubin, bootstrapped likelihood ratio test, and entropy model fit indices were used to find the best LCA model. The AIC imposes a lighter penalty on model complexity, tending to favor more complex models. In contrast, the BIC uses a stricter penalty and is generally regarded as more robust for determining the number of classes. The aBIC strikes a balance between AIC and BIC, providing an intermediate penalty that is particularly suitable for balanced model selection with moderate sample sizes. The Lo-Mendell-Rubin and bootstrapped likelihood ratio test statistically evaluate whether the current model demonstrates significant improvement over a model with one fewer class. A statistically significant *P* value from these tests indicates that the current model exhibits a substantially better fit compared to the simpler alternative model. An entropy value >0.8 indicates that 90% of individuals are correctly identified, whereas a value of 0.6 suggests that >20% are incorrectly classified. The best-fitting model is chosen based on a thorough evaluation of the aforementioned indicators and clinical interpretability.

#### Elastic Net

The elastic net (EN), proposed by Zou and Hastie [24], can eliminate characteristics unrelated to the dependent variable, such as least absolute shrinkage and selection operator regression, and choose a critical feature, such as ridge regression. By adjusting the parameters to balance the strength of the L1 and L2 regularization, an ideal model can be obtained. In this study, we used the EN (*glmnet* [25] and *ggplot2* [26] R packages) for feature selection and optimized the key parameters. A 10-fold cross-validation was used to prevent overfitting. The risk factor coefficients that were not closely related to multimorbidity were excluded by compressing them to 0, and the closely related possible risk factors that were not 0 were retained. The best and strict risk variables generally choose the lambda.1se model.

#### Multiple Logistic Regression

On the basis of the results of LCA and EN, multiple logistic regression (MLR) was used to identify the risk factors associated with different multimorbidity groups in the population with low CC. The statistical analysis was conducted using R (version 4.1.1; R Foundation for Statistical Computing) and Mplus (version 7.4; Muthén & Muthén). The regression study yielded data in the form of odds ratios accompanied by 95% CIs. The statistical significance criterion was determined using 2-sided *t* tests and a *P* value of .05.

## Results

### Descriptive Characteristics

**Figure 1.**
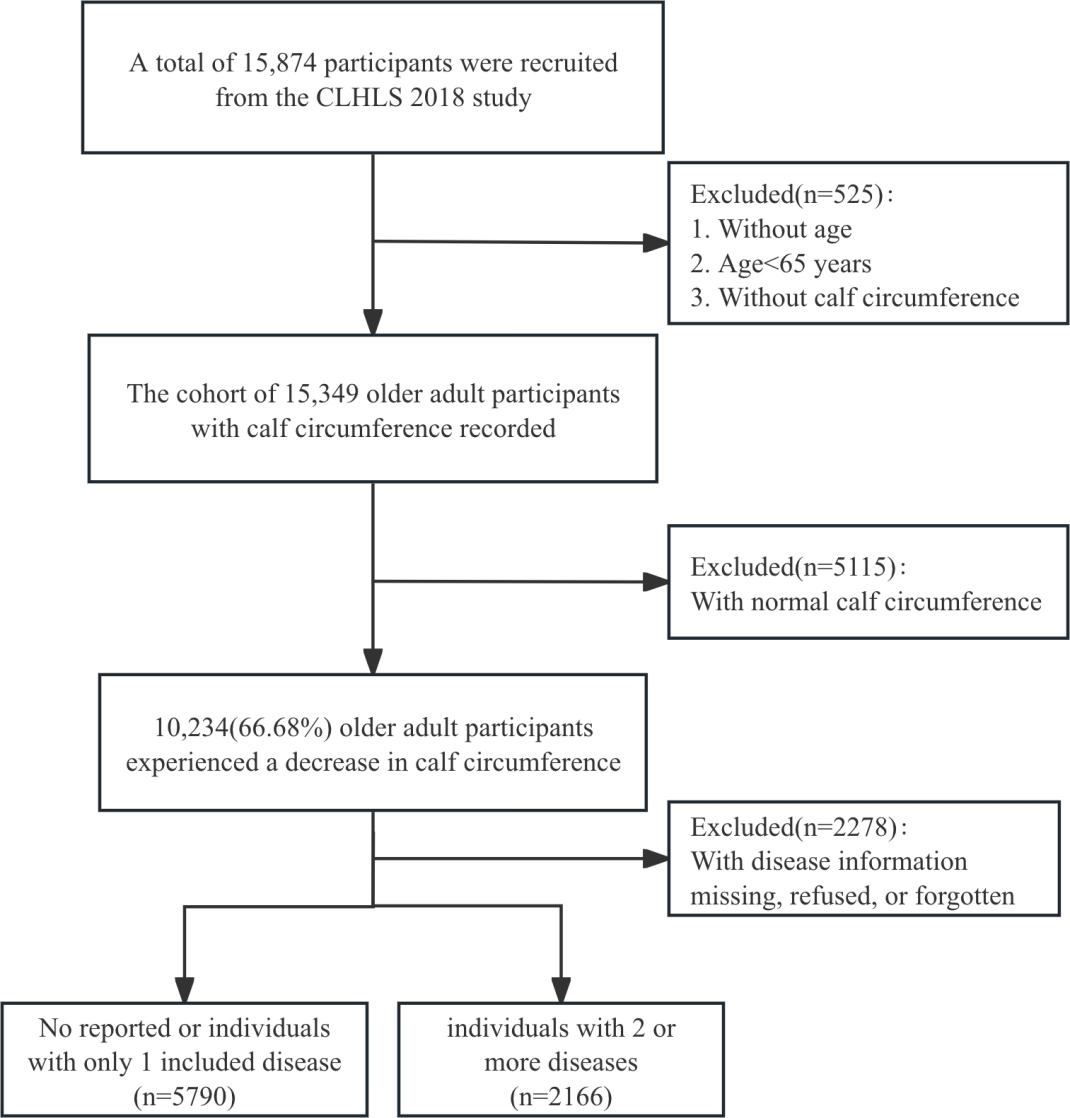
The flowchart of study design. CLHLS: Chinese Longitudinal Healthy Longevity Survey.

**Figure 2.**
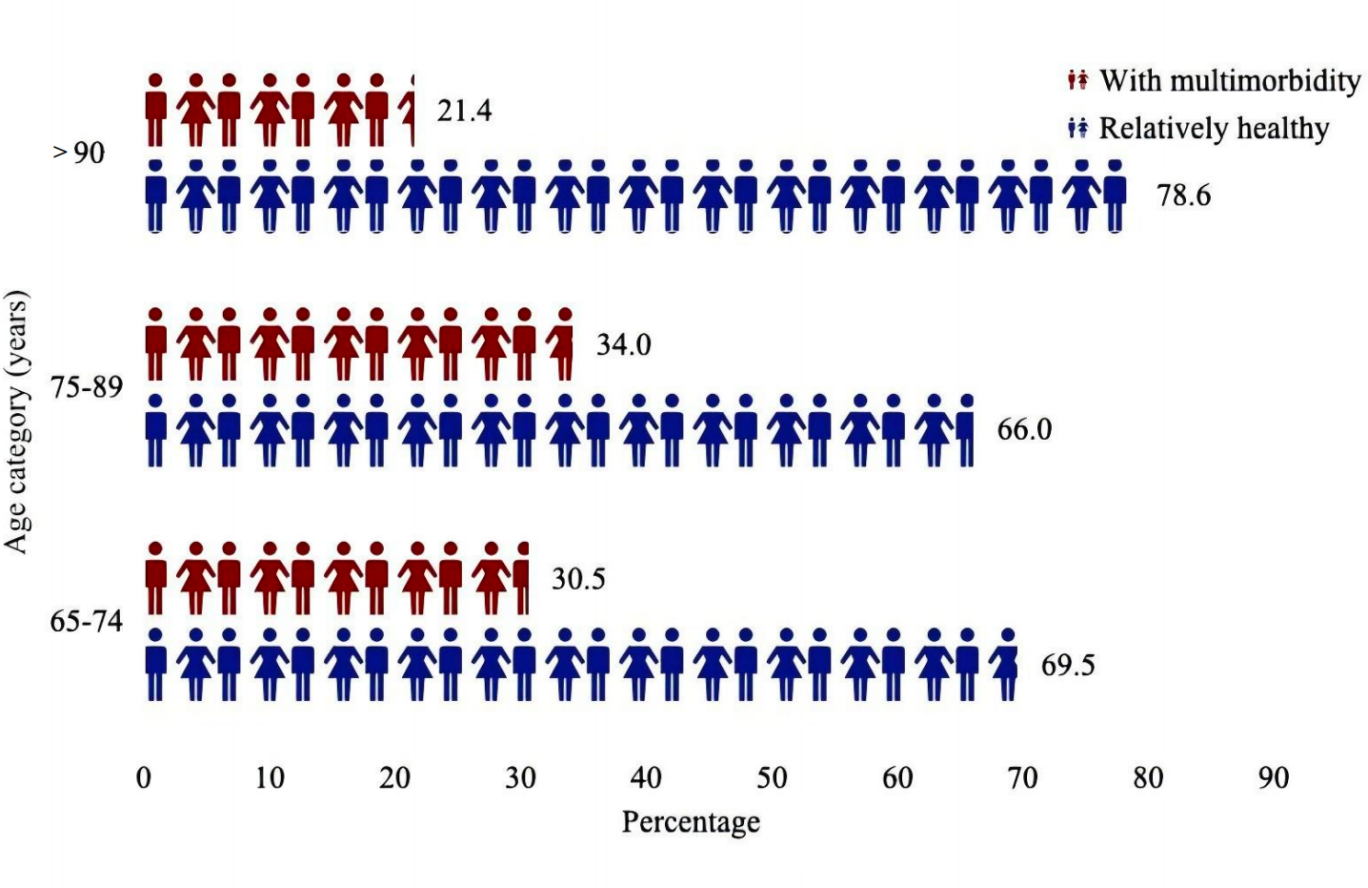
The distribution of multimorbidity in different age groups.

In this study, we included 7956 older adults with low CC (Multimedia Appendix 1; the detailed screening flow chart is shown in Figure 1). Their average age was 89 (range 79-99) years, with 49.77% (3960/7956) aged >90 years, and 39.19% (2269/5790) were men. Compared to the relatively healthy group, the multimorbidity group was younger, more likely to live in urban areas, more often partnered, and better educated. The multimorbidity group was likely to have a history of smoking and drinking habits (little or always). Over half of the people with multimorbidity dislike engaging in physical activities and sleep fairly or badly. Scores on the Mini-Mental State Examination, CES-D-10, GAD-7, and BMI were notably higher in the relatively healthy group. They may have greater difficulty with ADLs and overall health conditions. The proportions of distinct prevalent chronic diseases are shown in Table 1. The prevalence of multimorbidity increased between the ages of 65 and 89 years. However, after the age of 90 years, the proportion of multimorbidity decreased, and the majority of older adults were relatively healthy (Figure 2).

**Table 1.** The proportion of multimorbidity among older adult patients with low calf circumference (CC; N=7956).

Chronic diseases	Low CC, n (%)	Low CC and multimorbidity (n=2166), n (%)	Low CC and relatively healthy (n=5790), n (%)	*P* value
Hypertension	2787 (35.0)	1556 (71.8)	1231 (21.3)	<.001
Heart disease	1109 (13.9)	883 (40.8)	226 (3.9)	<.001
Bronchitis, emphysema, pneumonia, or asthma	819 (10.3)	506 (23.4)	313 (5.4)	<.001
Tuberculosis	61 (0.8)	49 (2.3)	12 (0.2)	<.001
Gastric or duodenal ulcer	384 (4.8)	292 (13.5)	92 (1.6)	<.001
Dyslipidemia	251 (3.2)	234 (10.8)	17 (0.3)	<.001
Cholecystitis or cholelith disease	282 (3.5)	224 (10.3)	58 (1.0)	<.001
Chronic nephritis	79 (1.0)	79 (3.7)	0 (0.0)	<.001
Hepatitis	27 (0.3)	21 (1.0)	6 (0.1)	<.001
Stroke or cardiovascular disease	735 (9.2)	576 (26.6)	159 (2.8)	<.001
Dementia	210 (2.6)	128 (5.9)	82 (1.4)	<.001
Epilepsy	22 (0.3)	20 (0.9)	2 (0.0)	<.001
Parkinson disease	58 (0.7)	45 (2.1)	13 (0.2)	<.001
Arthritis	735 (9.2)	590 (27.2)	145 (2.5)	<.001
Rheumatism or rheumatoid disease	360 (4.5)	294 (13.6)	66 (1.1)	<.001
Cancer	100 (1.3)	81 (3.7)	19 (0.3)	<.001
Diabetes	532 (6.7)	449 (20.7)	83 (1.4)	<.001

### Latent Class Analysis

**Figure 3.**
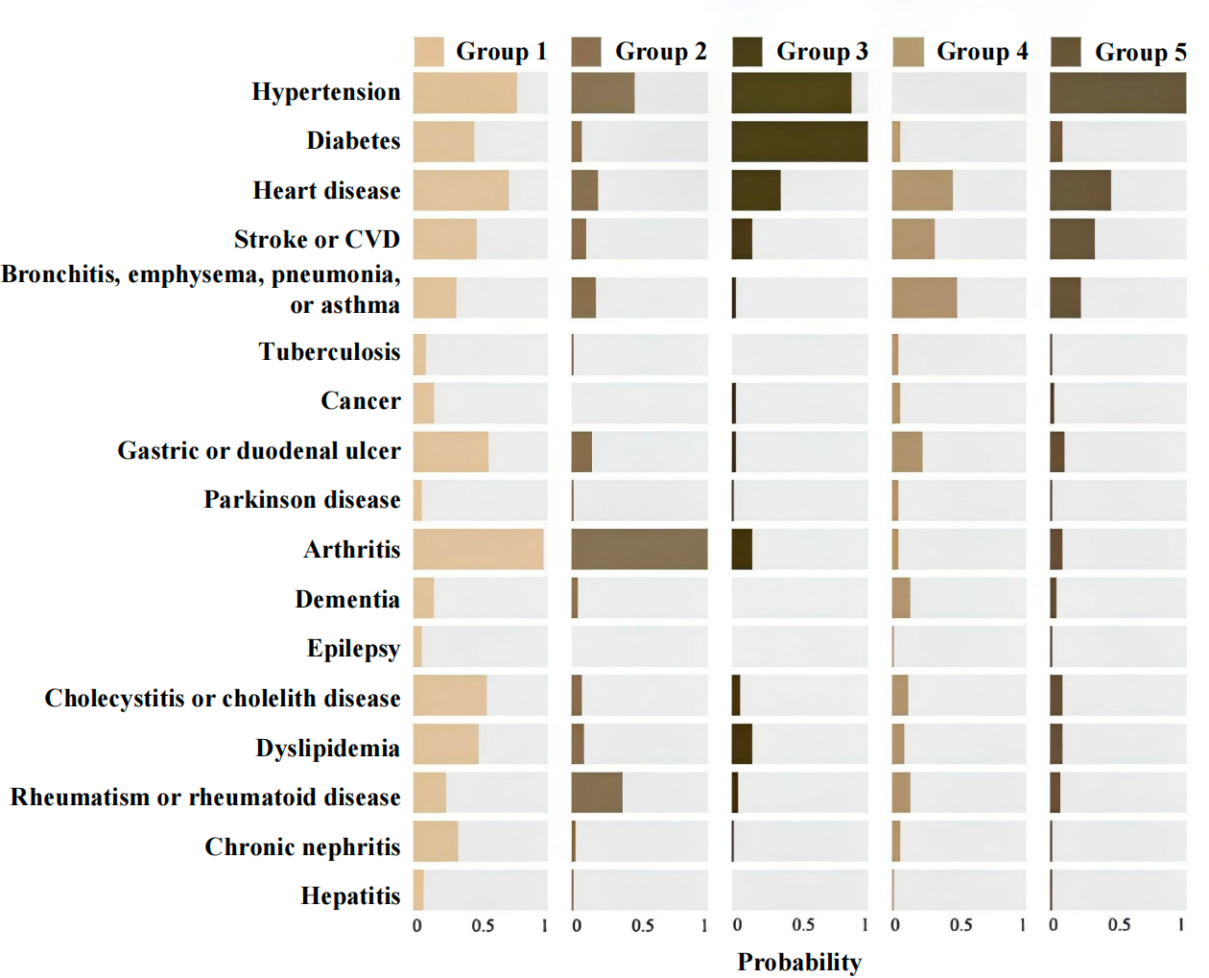
Cluster-specific conditional probabilities for the specific subgroups of older adults with low calf circumference and multimorbidity across 5 latent class models. CVD: cardiovascular disease.

Multimedia Appendix 2 compares fit statistics for models with 1 to 8 classes. AIC, BIC, and aBIC were low for class 5. Entropy (class 5=0.9) >0.8 suggests 90% correct categorization. In Figure 3, the membership probability is the “conditional prevalence” for each maintained class. We chose class 5 and named groups based on a combination of diseases and clinical evidence: multisystem morbidity diseases (group 1: 78/2166, 3.6%), arthritis-rheumatism or rheumatoid diseases (group 2: 400/2166, 18.47%), diabetes-hypertension diseases (group 3: 330/2166, 15.24%), respiratory-heart diseases (group 4: 347/2166, 16.02%), and CVDs (group 5: 1011/2166, 46.67%).

### Elastic Net

**Figure 4.**
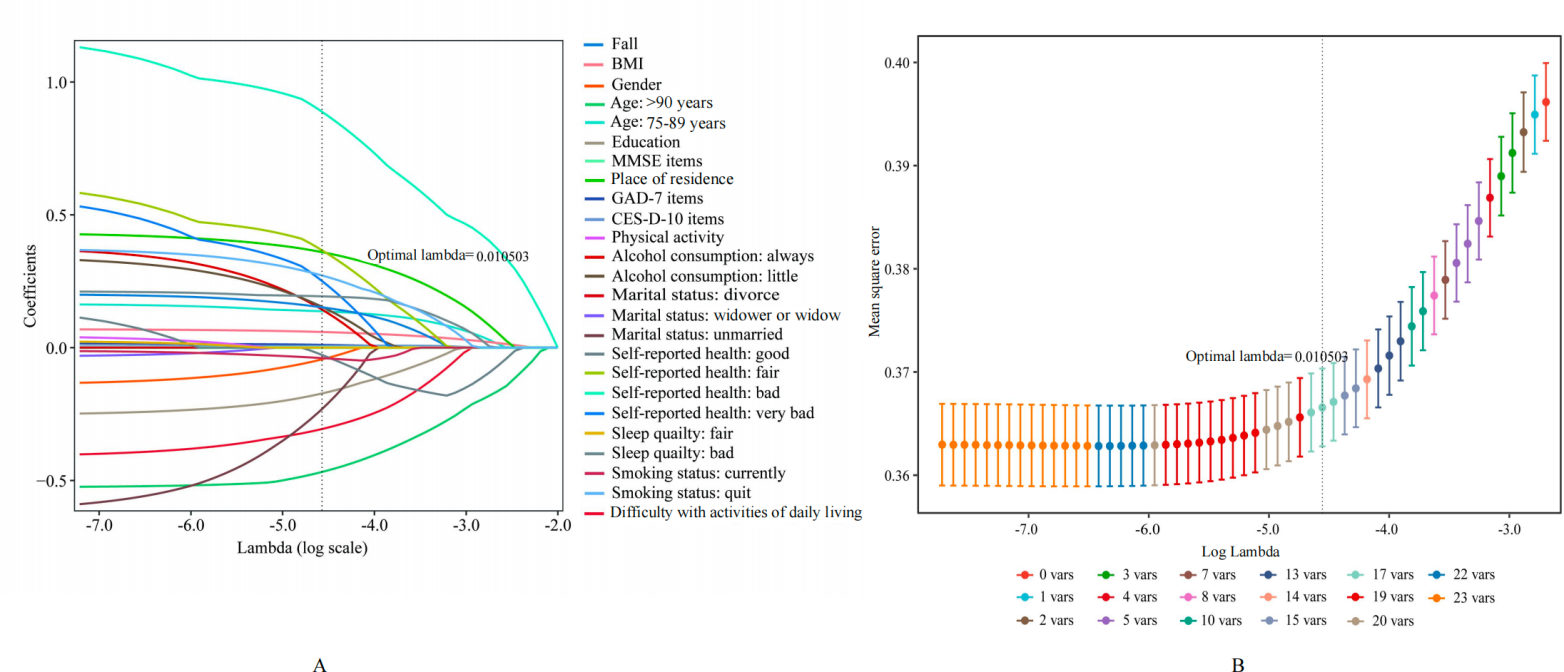
Plots for elastic net coefficients over different values of the penalty parameter. CES-D-10: Center for Epidemiological Studies-Depression Scale 10-item; GAD-7: Generalized Anxiety Disorder 7-item; MMSE: Mini-Mental State Examination.

The EN analysis included 25 variables, and the multicategorical variables were transformed using one-hot encoding (Figure 4A shows the process of variable selection as the penalty term increased). The best model was constructed using the 10-fold cross-validation approach, with α=.5 and λ with an SD of 0.010503 as the ideal parameters (Figure 4B shows the cross-validation plot for the penalty term). Finally, 12 strong risk variables and their association coefficients (Table 2) were obtained, including age, place of residence, education, difficulty with ADLs, smoking, current alcohol consumption, self-reported health, sleep quality, fall, BMI, CES-D-10, and GAD-7.

**Table 2.** The coefficient in the filter variables by the elastic net.

Variables	Coefficient
Age: >90 years	–0.43
Difficulty with activities of daily living	–0.25
Education	–0.10
Self-reported health: good	–0.09
Smoking status: currently	–0.03
CES-D-10^a^ items	0.00
GAD-7^b^ items	0.01
Current alcohol consumption: always	0.02
BMI	0.05
Self-reported health: very bad	0.07
Age: 75-89 years	0.10
Fall	0.11
Sleep quality: bad	0.18
Smoking status: quit	0.20
Self-reported health: fair	0.25
Place of residence	0.31
Self-reported health: bad	0.77

a CES-D-10: Center for Epidemiological Studies-Depression Scale 10-item.

b GAD-7: Generalized Anxiety Disorder 7-item.

### Multidimensional Risk Factors for Differences

The MLR identified risk variables for 5 latent categories, using the relatively healthy group as the reference class (Multimedia Appendix 3).

#### Demographic Data

The older adults aged 75 to 89 years were more likely to have CVDs than the relatively healthy group, whereas those aged >90 years were healthier. Urbanites had higher rates of multimorbidity, notably in the multisystem morbidity, diabetes-hypertension, CVDs, and respiratory-heart disease groups. Multisystem diseases, diabetes-hypertension, and CVDs are more common among educated older adults.

#### Behavioral Characteristics

Smoking history may enhance the chance of an older adult being diagnosed with respiratory-heart disease and CVDs. Current smoking does not appear to be a multimorbidity risk factor. Little and often alcohol use may be detrimental for diabetes-hypertension, CVDs, and respiratory-heart disease. Arthritis-rheumatism and CVDs were more common in older adults with poor sleep.

#### Physical and Psychological Health Characteristics

According to the MLR, multisystem morbidity increased the risk of depression. Assessment using the GAD-7, which evaluates anxiety symptoms over the preceding 2 weeks, indicated that the CVDs group may have had higher anxiety levels than the healthy group in the past 2 weeks. A higher BMI is a potential risk factor for falling across all multimorbidity groups, excluding respiratory-heart disease. Older adults with low CC who fell in the last year are more than twice as likely to develop arthritis-rheumatism or rheumatoid illness. The diabetes-hypertension, CVDs, and respiratory-heart disease groups were more likely to include older adults with ADL difficulties. The multimorbidity groups self-reported their health as fair, bad, or very bad.

## Discussion

### Principal Findings

In China, we found that low CC is a common phenomenon in community-dwelling older adults. LCA categorized these older patients into 5 groups: multisystem morbidity diseases, arthritis-rheumatism or rheumatoid diseases, diabetes-hypertension diseases, respiratory-heart diseases, and CVDs. MLR used 12 reliable risk variables established by EN and revealed that older adults with low CC and multimorbidity exhibited both comparable and dissimilar demographic, behavioral, and physical and psychological health characteristics.

Sarcopenia is a common geriatric syndrome characterized by the progressive loss of skeletal muscle mass and strength. It can be classified into primary sarcopenia, which is age related, and secondary sarcopenia, which is caused by factors such as malnutrition, physical inactivity, and chronic inflammation. The pathophysiology of sarcopenia involves disruptions in protein metabolism, neuromuscular degeneration, and abnormal fat infiltration, all of which are closely linked to the aging process [27,28]. Previous studies have demonstrated that CC, as a simple and feasible proxy for muscle mass, shows a declining trend with advancing age, with a notable inflection point occurring around 65 years. This pattern aligns with the natural progression of sarcopenia. Moreover, research suggests that calf muscle circumference, which was calculated by subtracting the calf subcutaneous fat thickness from the total CC, exhibits a stronger correlation with skeletal muscle mass, indicating its potential value as a more precise marker for early detection of sarcopenia. Given its ease of measurement in community and primary care settings, CC deserves further consideration as a practical screening tool. In addition, muscle atrophy associated with sarcopenia often coexists with bone mineral density loss and changes in lower limb morphology, which may indirectly influence CC measurements. Therefore, future studies should further investigate the multidimensional associations between CC, muscle mass, bone health, and functional status in older adults. In addition, there are chronic diseases such as arthritis, diabetes, varicose veins, neuromuscular diseases, and other diseases that can affect the CC. Prior research has established a correlation between low CC and chronic conditions such as hypertension and diabetes, as well as overall mortality [29,30]. Emerging evidence suggests that mitochondrial dysfunction in T cells may be a central mechanistic driver of multimorbidity. T cells exhibiting mitochondrial homeostatic imbalance synergistically exacerbate multiorgan pathophysiological cascades through chronic inflammatory activation, dysregulated metabolic interplay, and propagation of senescence-associated secretory phenotype signaling [31].

Our study identified CVDs as the most prevalent comorbidity pattern, providing a theoretical foundation for optimizing preventive care and therapeutic strategies. The marked disparity in CVD-related mortality between rural and urban areas in China (46.7% vs 44.3%) underscores the necessity for targeted implementation of mobile health programs in rural regions with limited health care resources [32]. The findings revealed that CC <33 cm is a risk factor for stroke and predisposes women to a higher risk of carotid plaque development [33]. After adjusting for potential confounders, a significant association between low CC and hypertension was identified. The underlying etiology of low CC may involve reduced capillary density and elevated resting heart rate [34]. Elevated heart rate is a consequential result of reduced stroke volume and reduced ability to tolerate exercise, which heightens the likelihood of cardiovascular incidents and perhaps leads to chronic hypertension. Integrating routine CC measurements into primary care screenings, coupled with patient education to enhance self-assessment awareness of modifiable risks such as resting heart rate and exercise tolerance, could mitigate the progression of CVDs in both populations.

Arthritis-rheumatism or rheumatoid disease is second only to CVDs as a comorbid pattern, which is consistent with the results of previous research [35]. Muscle atrophy is a common problem in joint diseases, such as osteoarthritis, and can also lead to articular muscle inhibition. Abnormal joint afferent nerves trigger the release of neurotransmitters at the spinal cord level, inhibiting the activity of α-motor neurons and resulting in decreased muscle activity, which leads to secondary muscle atrophy. A decrease in muscle measurement indicators, such as low CC, reflects reduced muscle cross-sectional area and increased fat infiltration, indicating muscle atrophy [36].

In this study, the combination of respiratory-heart disease was identified as the third most common comorbidity. Respiratory conditions such as chronic obstructive pulmonary disease or asthma can cause chronic hypoxemia, leading to pulmonary hypertension, increased right ventricular load, and eventually right heart dysfunction. Hypoxia also activates the sympathetic nervous system, elevating heart rate and blood pressure, which may further strain the cardiovascular system—potentially explaining this comorbidity pattern. Hypertension and diabetes commonly co-occur, largely due to overlapping pathophysiological mechanisms. Insulin resistance activates the sympathetic nervous system and the renin-angiotensin-aldosterone system, contributing to elevated blood pressure. Hyperglycemia accelerates endothelial dysfunction and atherosclerosis. Chronic inflammation, oxidative stress, and metabolic disturbances also play key roles in the shared development of both conditions. These comorbidity patterns have been established in separate investigations [37,38]. Our study sample consisted of older adults with low CC, which was a major source of discrepancy in comorbid patterns compared to other studies.

Our results confirmed the findings of prior studies, demonstrating that the prevalence of comorbidity was higher among younger older individuals than among those who lived longer [12]. One important source of potential bias is selection bias, particularly survivorship bias, which refers to the fact that individuals who reach exceptional longevity (eg, age ≥90 y) are more likely to possess inherent physiological resilience or to have consistently engaged in health-promoting behaviors, such as lifelong physical activity and balanced nutrition. Consequently, this population often appears as a cohort with low prevalence of comorbidities in observational studies. However, this phenomenon underscores a fundamental methodological limitation in aging research—namely, that the survival process inherently filters out individuals with greater vulnerability to chronic diseases, thereby potentially underestimating the true burden of morbidity in the general older adult population. Another factor that might explain this phenomenon is remembering bias: they are more prone to having both cognitive impairment and chronic diseases and may not accurately record their chronic conditions. There is a notable disparity in the prevalence of chronic diseases among older adults living in urban and rural areas in China, largely reflecting the unequal distribution of health care resources [39]. These differences may also arise from variations in lifestyle, health care access, and health awareness. Urban residents generally have better access to medical services but are more likely to engage in sedentary behavior and consume high-fat diets, increasing their risk of chronic conditions. In contrast, rural residents tend to engage in more physical activity but often face limited access to timely diagnosis and treatment. Socioeconomic and educational inequalities further contribute to these urban-rural disparities [12,40].

Smoking is widely recognized as an independent behavioral risk factor for respiratory diseases and CVDs [41]. In our cross-sectional analysis, we observed that personal smoking and drinking history were associated with increased likelihood of coexisting respiratory-heart diseases, CVDs, and diabetes-hypertension patterns in older adults. While these associations cannot establish causal relationships, they highlight potential modifiable lifestyle factors that warrant attention in older adults.

Rheumatoid arthritis, often accompanied by joint pain, swelling, and stiffness, may contribute to sleep disturbances, which are also commonly reported in older adults with multimorbidity. Moreover, both the disease itself and the side effects of medications such as nonsteroidal anti-inflammatory drugs and immunosuppressants may impair sleep quality [42]. Similarly, in individuals with CVDs (eg, coronary heart disease), nocturnal chest discomfort and medication use (eg, diuretics and beta-blockers) may further disrupt sleep. Importantly, joint dysfunction may lead to decreased physical activity and subsequent muscle disuse, potentially contributing to low CC. Conversely, muscle weakness related to sarcopenia may exacerbate joint instability and functional limitations [43]. Given the potential bidirectional relationship between them, clinicians should pay close attention to musculoskeletal function in older adults with joint diseases. Early identification of decreased lower limb muscle mass in patients with arthritis may help prevent further functional decline.

Regarding physical and psychological health characteristics, one study confirmed that people with multiple illnesses were 2 to 3 times more prone to developing depression than those without multimorbidity. Our findings indicate that older adults with multimorbidity are more likely to report psychological distress, including depressive and anxiety symptoms, consistent with prior cross-sectional and longitudinal studies [44]. A survey reported that the severity of anxiety symptoms is linked to a higher risk of cardiovascular problems [45]. Although the directionality of these associations remains uncertain, the co-occurrence of mental and physical health conditions underscores the need for integrated care. Obesity is a common risk factor for several diseases, and an observational study found that a higher BMI is one of the risk factors for complex and frequent morbidity [46]. Arthritis-rheumatoid joint diseases may cause calf muscle atrophy and lower limb muscle weakness, increasing the likelihood of falls [47]. Consistent with the existing evidence, loss of ADLs is a risk factor for multiple morbidities in older adults [48]. Higher body weight, joint disorders, and reduced physical function suggest that clinical efforts in older adults should focus on weight management, appropriate rehabilitation or strength training to maintain musculoskeletal health, and regular functional assessments. These measures may help identify high-risk individuals early and facilitate timely interventions to improve overall health outcomes.

Self-rated health, as a simple and cost-effective measure, was associated with multimorbidity in our study. Older adults with negative perceptions of their health may be more likely to present with multiple chronic conditions. Studies conducted in several countries have similarly supported this finding, showing that patients with 3 or 4 disorders are significantly more likely to report poor health [49]. This highlights the potential value of incorporating self-rated health into routine assessments to help identify individuals at higher health risk.

### Strengths and Limitations

Our study has several strengths. First, our results are more applicable because we used a sizable cohort of long-lived Chinese citizens, which is nationally representative. Second, the EN develops a variable model specifically suited to this population, identifying significant risk factors. Third, due to the potential existence of mixed factors in the study population, we did not simply use the disease classification method of combining 2, 3, or more diseases; instead, we selected LCA for analysis to obtain different representative comorbidity groups. More significantly, this is the first study to look at risk factors for multimorbidity classifications in older adults with low CC, which has significant ramifications for clinical practice and health policy.

Our study also has some limitations. Although it was not feasible to draw firm conclusions about causality from the cross-sectional study, EN allowed us to develop a trustworthy risk model. According to studies, CC measurements in individuals with lower limb edema yielded erroneous findings, and some patients with heart failure were excluded from the study due to their swollen calves. Noncommunicable illnesses are currently China’s top cause of death for older adults. Some infectious illnesses have high fatality rates, which might be the reason we did not find any evidence of their prevalence.

### Conclusions

This study found that individuals with low CC and multimorbidity constituted a large group that cannot be ignored in the older adult population of the Chinese community, and potential risk factors were revealed in different combinations of multimorbidity. These findings underscore the imperative to operationalize precision public health frameworks—integrating geriatric biomarkers such as low CC into multidisciplinary care models—to mitigate adverse outcomes in high-risk older adults. Public health efforts should prioritize community-based interventions such as chronic disease self-management, coordinated primary care, and preventive lifestyle support. Future research is needed to evaluate the effectiveness and scalability of such approaches to reduce the burden of multimorbidity and promote healthy aging.

## Supplementary material

10.2196/68760 Multimedia Appendix 1 Characteristics of older adults with low calf circumference and multimorbidity.

10.2196/68760 Multimedia Appendix 2 Comparison of model fit statistics for latent class analysis across 1 to 8 classes.

10.2196/68760 Multimedia Appendix 3 Multidimensional risk factors associated with different groups.

## Abbreviations

aBIC: adjusted Bayesian information criterion
ADL: activity of daily living
AIC: Akaike information criterion
BIC: Bayesian information criterion
CC: calf circumference
CES-D-10: Center for Epidemiological Studies-Depression Scale 10-item
CLHLS: Chinese Longitudinal Healthy Longevity Survey
CVD: cardiovascular disease
EN: elastic net
GAD-7: Generalized Anxiety Disorder 7-item
LCA: latent class analysis
MLR: multiple logistic regression

## References

[1] Cruz-Jentoft AJ, Landi F, Topinková E, Michel JP (2010). Understanding sarcopenia as a geriatric syndrome. Curr Opin Clin Nutr Metab Care.

[2] Papadopoulou SK (2020). Sarcopenia: a contemporary health problem among older adult populations. Nutrients.

[3] Hwang AC, Liu LK, Lee WJ, Peng LN, Chen LK (2018). Calf circumference as a screening instrument for appendicular muscle mass measurement. J Am Med Dir Assoc.

[4] Wang X, Yang F, Zhu M (2023). Development and assessment of assisted diagnosis models using machine learning for identifying elderly patients with malnutrition: cohort study. J Med Internet Res.

[5] Borges K, Artacho R, Jodar-Graus R, Molina-Montes E, Ruiz-López MD (2022). Calf circumference, a valuable tool to predict sarcopenia in older people hospitalized with hip fracture. Nutrients.

[6] Wu SE, Chen WL (2022). Calf circumference refines sarcopenia in correlating with mortality risk. Age Ageing.

[7] Pérez-Zepeda MU, Gutiérrez-Robledo LM (2016). Calf circumference predicts mobility disability: a secondary analysis of the Mexican health and ageing study. Eur Geriatr Med.

[8] Tsai AC, Chang TL (2011). The effectiveness of BMI, calf circumference and mid-arm circumference in predicting subsequent mortality risk in elderly Taiwanese. Br J Nutr.

[9] Wu CJ, Kao TW, Lin YY (2017). Examining the association between anthropometric parameters and telomere length and mortality risk. Oncotarget.

[10] Bellantuono I (2018). Find drugs that delay many diseases of old age. Nature New Biol.

[11] Sum G, Hone T, Atun R (2018). Multimorbidity and out-of-pocket expenditure on medicines: a systematic review. BMJ Glob Health.

[12] Yao SS, Cao GY, Han L (2020). Prevalence and patterns of multimorbidity in a nationally representative sample of older Chinese: results from the China health and retirement longitudinal study. J Gerontol A Biol Sci Med Sci.

[13] Olson JE, Takahashi PY, St Sauver JM (2018). Understanding the patterns of multimorbidity. Mayo Clin Proc.

[14] Dodds RM, Granic A, Robinson SM, Sayer AA (2020). Sarcopenia, long-term conditions, and multimorbidity: findings from UK Biobank participants. J Cachexia Sarcopenia Muscle.

[15] Yang K, Yang S, Chen Y (2023). Multimorbidity patterns and associations with gait, balance and lower extremity muscle function in the elderly: a cross-sectional study in Northwest China. Int J Gen Med.

[16] Kawakami R, Murakami H, Sanada K (2015). Calf circumference as a surrogate marker of muscle mass for diagnosing sarcopenia in Japanese men and women. Geriatr Gerontol Int.

[17] Chen LK, Woo J, Assantachai P (2020). Asian Working Group for Sarcopenia: 2019 consensus update on sarcopenia diagnosis and treatment. J Am Med Dir Assoc.

[18] Chen ZT, Wang XM, Zhong YS, Zhong WF, Song WQ, Wu XB (2024). Association of changes in waist circumference, waist-to-height ratio and weight-adjusted-waist index with multimorbidity among older Chinese adults: results from the Chinese longitudinal healthy longevity survey (CLHLS). BMC Public Health.

[19] Sun X, Liu X, Wang X, Pang C, Yin Z, Zang S (2024). Association between residential proximity to major roadways and chronic multimorbidity among Chinese older adults: a nationwide cross-sectional study. BMC Geriatr.

[20] Skou ST, Mair FS, Fortin M (2022). Multimorbidity. Nat Rev Dis Primers.

[21] Yuan S, Larsson SC (2023). Epidemiology of sarcopenia: prevalence, risk factors, and consequences. Metab Clin Exp.

[22] Cheng L, Sit JWH, Chan HYL (2021). Sarcopenia risk and associated factors among Chinese community-dwelling older adults living alone. Sci Rep.

[23] Stekhoven DJ, Bühlmann P (2012). MissForest--non-parametric missing value imputation for mixed-type data. Bioinformatics.

[24] Zou H, Hastie T (2005). Regularization and variable selection via the elastic net. J R Stat Soc Series B Stat Methodol.

[25] Engebretsen S, Bohlin J (2019). Statistical predictions with glmnet. Clin Epigenetics.

[26] Ginestet C (2011). ggplot2: elegant graphics for data analysis. J R Stat Soc Ser A Stat Soc.

[27] Pedersen BK, Febbraio MA (2012). Muscles, exercise and obesity: skeletal muscle as a secretory organ. Nat Rev Endocrinol.

[28] Nelke C, Dziewas R, Minnerup J, Meuth SG, Ruck T (2019). Skeletal muscle as potential central link between sarcopenia and immune senescence. EBioMedicine.

[29] Patiño-Villada FA, Estrada-Restrepo A, Aristizábal J (2023). Handgrip strength in older adults from Antioquia-Colombia and comparison of cutoff points for dynapenia. Sci Rep.

[30] Wang X, Ying Y, Pei M (2023). Calf circumference change and all-cause mortality among community-dwelling Chinese older people. Clin Nutr.

[31] Desdín-Micó G, Soto-Heredero G, Aranda JF (2020). T cells with dysfunctional mitochondria induce multimorbidity and premature senescence. Science.

[32] Hu SS, The Writing Committee Of The Report On Cardiovascular Health And Diseases In China (2023). Report on cardiovascular health and diseases in China 2021: an updated summary. J Geriatr Cardiol.

[33] Debette S, Leone N, Courbon D (2008). Calf circumference is inversely associated with carotid plaques. Stroke.

[34] Hedman A, Andersson PE, Reneland R, Lithell HO (2001). Insulin-mediated changes in leg blood flow are coupled to capillary density in skeletal muscle in healthy 70-year-old men. Metab Clin Exp.

[35] Zhang R, Lu Y, Shi L, Zhang S, Chang F (2019). Prevalence and patterns of multimorbidity among the elderly in China: a cross-sectional study using national survey data. BMJ Open.

[36] Wiewiorski M, Dopke K, Steiger C, Valderrabano V (2012). Muscular atrophy of the lower leg in unilateral post traumatic osteoarthritis of the ankle joint. Int Orthop.

[37] Zhao X, Zhang Q, Ma C, Liu H, Chen Y (2023). Association between multimorbidity patterns and healthcare costs among middle-aged and older adults in China. Arch Gerontol Geriatr.

[38] Zhang Q, Han X, Zhao X, Wang Y (2022). Multimorbidity patterns and associated factors in older Chinese: results from the China health and retirement longitudinal study. BMC Geriatr.

[39] Zhang X, Dupre ME, Qiu L, Zhou W, Zhao Y, Gu D (2017). Urban-rural differences in the association between access to healthcare and health outcomes among older adults in China. BMC Geriatr.

[40] Bonnell LN, Clifton J, Rose GL, Waddell EN, Littenberg B (2022). Urban-rural differences in mental and physical health among primary care patients with multiple chronic conditions: a secondary analysis from a randomized clinical trial. Int J Environ Res Public Health.

[41] Freund O, Shetrit A, Bar-Shai A (2024). Smoking and respiratory diseases in patients with coronary microvascular dysfunction. Am J Med.

[42] Fitzcharles MA, Lussier D, Shir Y (2010). Management of chronic arthritis pain in the elderly. Drugs Aging.

[43] Prado CM, Landi F, Chew STH (2022). Advances in muscle health and nutrition: a toolkit for healthcare professionals. Clin Nutr.

[44] Read JR, Sharpe L, Modini M, Dear BF (2017). Multimorbidity and depression: a systematic review and meta-analysis. J Affect Disord.

[45] van Zutphen EM, Kok AAL, Muller M (2023). Cardiovascular risk indicators among depressed persons: a special case?. J Affect Disord.

[46] Kivimäki M, Strandberg T, Pentti J (2022). Body-mass index and risk of obesity-related complex multimorbidity: an observational multicohort study. Lancet Diabetes Endocrinol.

[47] Bennett JL, Pratt AG, Dodds R, Sayer AA, Isaacs JD (2023). Rheumatoid sarcopenia: loss of skeletal muscle strength and mass in rheumatoid arthritis. Nat Rev Rheumatol.

[48] Sieber S, Roquet A, Lampraki C, Jopp DS (2023). Multimorbidity and quality of life: the mediating role of ADL, IADL, loneliness, and depressive symptoms. Innov Aging.

[49] Ishizaki T, Kobayashi E, Fukaya T, Takahashi Y, Shinkai S, Liang J (2019). Association of physical performance and self-rated health with multimorbidity among older adults: results from a nationwide survey in Japan. Arch Gerontol Geriatr.

