# Supplementary material for: Exploring Risk Factors Related to Low Calf Circumference in Older Adults With Multimorbidity: Cross-Sectional Latent Class Analysis
Source: JMIR Aging. 2025 Oct 2;8:e68760. doi: 10.2196/68760 (PMC12490777; doi:10.2196/68760)
Supplement: Multimedia Appendix 1 [file aging-v8-e68760-s001.docx]

Multimedia Appendix 1. Characteristics of older adults with low calf circumference and multimorbidity.

| Variables | Definition | Total (N=7956) | Multimorbidity condition | | *P* value |
| --- | --- | --- | --- | --- | --- |
|  |  |  | With multimorbidity  (N=2166) | Relatively healthy  (N=5790) |  |
| Demographic Data | | | | | |
| Age [years, Median (Q1^a^, Q3^b^)] |  | 89 (79-99) | 86 (78-94) | 90 (80-100) | <.001 |
| Age category (years) | 65-74 | 1157 (14.5) | 353 (16.3) | 804 (13.9) | <.001 |
|  | 75-89 | 2839 (35.7) | 966 (44.6) | 1873 (32.4) |  |
|  | >90 | 3960 (49.8) | 847 (39.1) | 3113 (53.8) |  |
| Gender [n (%)] | man | 3121 (39.2) | 852 (39.3) | 2269 (39.2) | .905 |
|  | woman | 4835 (60.78) | 1314 (60.7) | 3521 (60.8) |  |
| Place of residence [n (%)] | urban area | 4196 (52.7) | 1307 (60.3) | 2889 (49.9) | <.001 |
|  | rural area | 3760 (47.3) | 859 (39.7) | 2901 (50.1) |  |
| Marital status [n (%)] | married | 2705 (34.0) | 850 (39.2) | 1855 (32.0) | <.001 |
|  | divorced | 28 (0.4) | 9 (0.4) | 19 (0.3) |  |
|  | widower/widow | 5155 (64.8) | 1293 (59.7) | 3862 (66.7) |  |
|  | unmarried | 68 (0.9) | 14 (0.7) | 54 (0.9) |  |
| Education [n (%)] | literacy | 4111 (51.7) | 1248 (57.6) | 2863 (49.5) | <.001 |
|  | illiteracy | 3845 (48.3) | 918 (42.4) | 2927 (50.6) |  |
| Behavioral Characteristics | | | | | |
| Smoking status [n (%)] | never | 5727 (72.0) | 1525 (70.4) | 4202 (72.6) | <.001 |
|  | currently | 1155 (14.5) | 269 (12.4) | 886 (15.3) |  |
|  | quit | 1074 (13.5) | 372 (17.2) | 702 (12.1) |  |
| Alcohol consumption currently [n (%)] | never | 6079 (76.4) | 1204 (55.6) | 4296 (74.2) | <.001 |
|  | little | 864 (10.9) | 274 (12.7) | 590 (10.2) |  |
|  | always | 1013 (12.7) | 688 (31.8) | 804 (13.9) |  |
| Physical activity [n (%)] | yes | 3754 (47.2) | 1066 (49.2) | 2688 (46.4) | .026 |
|  | no | 4202 (52.8) | 1100 (50.8) | 3102 (53.6) |  |
| Sleep quailty [n (%)] | good | 3856 (48.5) | 920 (42.5) | 2936 (50.7) | <.001 |
|  | fair | 2755 (34.6) | 763 (35.2) | 1992 (34.4) |  |
|  | bad | 1345 (16.9) | 483 (22.3) | 862 (14.9) |  |
| Physical And Psychological Health Characteristics | | | | | |
| MMSE items^c^ [socre, Median (Q1^a^, Q3^b^)] |  | 26.0 (23.3-28.2) | 26.2 (23.7-29.0) | 26.0 (23.1-28.1） | .027 |
| CESD-10 items^d^ [socre, Median (Q1^a^, Q3^b^)] | | 6.5 (4.0-9.0) | 7.0 (4.4-10.0) | 6.2 (4.0-9.0) | <.001 |
| GAD-7 items^e^ [socre, Median (Q1^a^, Q3^b^)] |  | 0.0 (0.0-1.9) | 0.0 (0.0-2.0) | 0.0 (0.0-1.1) | <.001 |
| BMI^f^ [kg/m^2^, Median (Q1^a^, Q3^b^)] |  | 20.7 (18.7-23.0) | 21.5 (19.2-23.9) | 20.4 (18.5-22.7) | <.001 |
| Fall [n (%)] | yes | 1913 (24.0) | 602 (27.8) | 1311 (22.6) | <.001 |
|  | no | 5635 (70.8) | 1156 (534) | 4479 (77.3) |  |
| Difficulty with activities of daily living [n (%)] | yes | 2582 (32.5) | 774 (35.7) | 1808 (31.2) | <.001 |
|  | no | 5374 (67.6) | 1392 (64.3) | 3982 (68.8) |  |
| Self-reported health [n (%)] | very good | 843 (10.6) | 150 (6.9) | 693 (12.0) | <.001 |
|  | good | 2602 (32.7) | 527 (24.3) | 2075 (35.8) |  |
|  | fair | 3195 (40.2) | 938 (43.3) | 2257 (39.0) |  |
|  | poor | 1002 (12.6) | 451 (20.8) | 551 (9.5) |  |
|  | very poor | 314 (4.0) | 100 (4.6) | 214 (3.7) |  |
| ^a^Q1, first quartile.  ^b^Q3, third quartile.  ^c^MMSE, Mini-Mental State Examination.  ^d^CES-D-10, Center for Epidemiological Studies-Depression Scale 10-item.  ^e^GAD-7, Generalized Anxiety Disorder 7-item.  ^f^BMI, Body Mass Index. | | | | | |
|  | | | | | |
