# Supplementary material for: Exploring Risk Factors Related to Low Calf Circumference in Older Adults With Multimorbidity: Cross-Sectional Latent Class Analysis
Source: JMIR Aging. 2025 Oct 2;8:e68760. doi: 10.2196/68760 (PMC12490777; doi:10.2196/68760)
Supplement: Multimedia Appendix 2 [file aging-v8-e68760-s002.docx]

Multimedia Appendix 2. Comparison of model fit statistics for latent class analysis across 1 to 8 classes.

| Model | | k^a^ | AIC^b^ | BIC^c^ | aBIC^d^ | Entropy | Lo-Mendell-Rubin | bootstrapped likelihood ratio test |
| --- | --- | --- | --- | --- | --- | --- | --- | --- |
| class1 | | 17 | 25218.0 | 25314.6 | 25260.6 | — | — | — |
| class2 | | 35 | 24812.7 | 25011.5 | 24900.3 | 0.5 | 0.0 | 0.0 |
| class3 | | 53 | 24643.3 | 24944.4 | 24776.0 | 0.7 | 0.1 | 0.0 |
| class4 | | 71 | 24433.7 | 24837.1 | 24611.5 | 0.8 | 0.0 | 0.2 |
| class5 | | 89 | 24348.5 | 24854.1 | 24571.4 | 0.9 | 0.0 | 0.0 |
| class6 | | 107 | 24272.6 | 24880.5 | 24540.5 | 0.8 | 0.2 | 0.0 |
| class7 | | 125 | 24200.9 | 24911.0 | 24513.9 | 0.8 | 0.1 | 0.0 |
| class8 | | 143 | 24088.3 | 24900.6 | 24446.3 | 0.8 | 0.5 | 0.0 |
| ^a^k, Number of Free Parameters.  ^b^AIC, Akaike Information Criterion.  ^c^BIC, Bayesian Information Criterion.  ^d^aBIC, Adjusted Bayesian Information Criterion. | | | | | | | | |
|  |  |  |  |  |  |  |  |  |
