# Supplementary material for: Exploring Risk Factors Related to Low Calf Circumference in Older Adults With Multimorbidity: Cross-Sectional Latent Class Analysis
Source: JMIR Aging. 2025 Oct 2;8:e68760. doi: 10.2196/68760 (PMC12490777; doi:10.2196/68760)
Supplement: Multimedia Appendix 3 [file aging-v8-e68760-s003.docx]

Multimedia Appendix 3. Multidimensional risk factors associated with different groups.

| Independent variables | Relative healthy group (n=5790) | Multisystem morbidity diseases group (n=78) | | Arthritis-rheumatism or rheumatoid diseases group (n=400) | | Diabetes-hypertension diseases group (n=330) | | Respiratory-heart diseases group (n=347) | | Cardiovascular diseases (CVDs) group (n=1011) | |
| --- | --- | --- | --- | --- | --- | --- | --- | --- | --- | --- | --- |
|  | (Ref) | OR (95% CI) | *P* value | OR (95% CI) | *P* value | OR (95% CI) | *P* value | OR (95% CI) | *P* value | OR (95% CI) | *P* value |
| Demographic Data | | | | | | | | | | | |
| Age category (ref = 65-74, years） | |  |  |  |  |  |  |  |  |  |  |
| 75-89 | 1.00 | 1.52 (0.82 - 2.82) | .018 | 0.98 (0.72 - 1.32) | .873 | 0.96 (0.71 - 1.29) | .785 | 0.99 (0.70 - 1.39) | .949 | 1.38 (1.12 - 1.71) | .003 |
| >90 | 1.00 | 0.38 (0.17 - 0.83) | .016 | 0.66 (0.48 - 0.92) | .014 | 0.32 (0.23 - 0.47) | < .001 | 0.53 (0.36 - 0.76) | .001 | 0.67 (0.53 - 0.84) | .001 |
| Urban area (ref = rural) | 1.00 | 3.83 (2.20 - 6.65) | < .001 | 1.22 (0.99 - 1.50) | .061 | 2.42 (1.89 - 3.10) | < .001 | 1.43 (1.14 - 1.78) | .002 | 1.45 (1.26 - 1.66) | < .001 |
| Illiteracy (ref = literacy) | 1.00 | 0.28 (0.16 - 0.49) | < .001 | 0.82 (0.66 - 1.03) | .087 | 0.71 (0.55 - 0.91) | .007 | 0.84 (0.66 - 1.06) | .144 | 0.86 (0.74 - 0.99) | .041 |
| Behavioral Characteristics | | | | | | | | | | | |
| Smoking status (ref = never) | |  |  |  |  |  |  |  |  |  |  |
| Currently | 1.00 | 0.27 (0.08 - 0.89) | .032 | 0.95 (0.69 - 1.32) | .770 | 0.63 (0.41 - 0.95) | .029 | 1.10 (0.78 - 1.57) | .582 | 1.02 (0.82 - 1.27) | .852 |
| Quit | 1.00 | 0.76 (0.35 - 1.69) | .504 | 1.01 (0.72 - 1.42) | .936 | 1.38 (0.98 - 1.93) | .063 | 1.68 (1.23 - 2.29) | .001 | 1.47 (1.20 - 1.81) | < .001 |
| Alcohol consumption currently (ref =never) | | |  |  |  |  |  |  |  |  |  |
| Little | 1.00 | 0.59 (0.14 - 2.59) | .488 | 0.92 (0.59 - 1.45) | .721 | 1.50 (0.87 - 2.57) | .144 | 1.66 (1.05 - 2.63) | .031 | 1.66 (1.24 - 2.23) | .001 |
| Always | 1.00 | 1.92 (0.74 - 5.03) | .182 | 1.13 (0.81 - 1.57) | .489 | 1.84 (1.19 - 2.86) | .006 | 1.36 (0.92 - 2.03) | .128 | 1.50 (1.17 - 1.91) | .001 |
| Sleep quailty (ref = good) |  |  |  |  |  |  |  |  |  |  |  |
| Fair | 1.00 | 1.14 (0.64 - 2.01) | .664 | 1.12 (0.88 - 1.44) | .357 | 0.86 (0.65 - 1.13) | .286 | 0.94 (0.71 - 1.23) | .648 | 1.08 (0.91 - 1.27) | .393 |
| Bad | 1.00 | 0.99 (0.51 - 1.95) | .986 | 1.39 (1.02 - 1.89) | .040 | 1.07 (0.75 - 1.52) | .716 | 1.28 (0.92 - 1.79) | .138 | 1.28 (1.03 - 1.59) | .024 |
| Physical And Psychological Health Characteristics | | | | | | | | | | | |
| CES-D-10 items^a^ | 1.00 | 1.07 (1.00 - 1.15) | .047 | 1.01 (0.97 - 1.04) | .709 | 0.99 (0.96 - 1.03) | .712 | 1.01 (0.97 - 1.04) | .735 | 0.99 (0.97 - 1.02) | .635 |
| GAD-7 items^b^ | 1.00 | 1.01 (0.93 - 1.09) | .806 | 1.02 (0.98 - 1.06) | .414 | 0.98 (0.93 - 1.04) | .521 | 1.00 (0.96 - 1.05) | .920 | 1.03 (1.00 - 1.06) | .041 |
| BMI^c^ | 1.00 | 1.13 (1.07 - 1.20) | < .001 | 1.05 (1.02 - 1.08) | .002 | 1.15 (1.12 - 1.18) | < .001 | 0.99 (0.96 - 1.02) | .484 | 1.08 (1.06 - 1.10) | < .001 |
| Fall (ref = no) | 1.00 | 1.61 (0.97 - 2.67) | .066 | 1.60 (1.27 - 2.02) | < .001 | 1.15 (0.87 - 1.51) | .333 | 1.18 (0.91 - 1.52) | .208 | 1.13 (0.96 - 1.33) | .146 |
| Difficulty with activities of daily living (ref = yes) | 1.00 | 0.98 (0.55 - 1.74) | .941 | 1.03 (0.80 - 1.32) | .842 | 0.68 (0.52 - 0.91) | .008 | 0.43 (0.34 - 0.56) | < .001 | 0.65 (0.55 - 0.77) | < .001 |
| Self-reported health (ref = very good) | | |  |  |  |  |  |  |  |  |  |
| Good | 1.00 | 0.74 (0.25 - 2.15) | .575 | 1.26 (0.82 - 1.95) | .294 | 1.39 (0.84 - 2.28) | .200 | 0.75 (0.48 - 1.17) | .200 | 1.33 (1.00 - 1.77) | .054 |
| Fair | 1.00 | 1.23 (0.45 - 3.36) | .688 | 1.81 (1.18 - 2.78) | .007 | 2.74 (1.69 - 4.46) | < .001 | 1.31 (0.86 - 2.00) | .209 | 2.01 (1.52 - 2.67) | < .001 |
| Bad | 1.00 | 6.26 (2.24 - 17.46) | < .001 | 3.22 (1.99 - 5.21) | < .001 | 4.47 (2.59 - 7.73) | < .001 | 2.73 (1.71 - 4.35) | < .001 | 2.90 (2.09 - 4.02) | < .001 |
| Very bad | 1.00 | 4.33 (1.10 - 17.04) | .036 | 1.17 (0.56 - 2.43) | .680 | 2.36 (1.04 - 5.33) | .039 | 1.36 (0.72 - 2.57) | .351 | 1.95 (1.25 - 3.04) | .003 |
| ^a^CES-D-10,Center for Epidemiological Studies-Depression Scale 10-item.  ^b^GAD-7, Generalized Anxiety Disorder 7-item.  ^c^BMI, Body Mass Index. | | | | | | | | | | | |
